# Bidirectional and Cross-Hemispheric Modulations of Face-Selective Neural Activity Induced by Electrical Stimulation within the Human Cortical Face Network

**DOI:** 10.3390/brainsci14090906

**Published:** 2024-09-06

**Authors:** Luna Angelini, Corentin Jacques, Louis Maillard, Sophie Colnat-Coulbois, Bruno Rossion, Jacques Jonas

**Affiliations:** 1 Université de Lorraine, IMoPA, UMR CNRS 7365, F-54000 Nancy, France; luna.angelini@univ-lorraine.fr (L.A.);; 2 Université de Lorraine, CHRU-Nancy, Service de Neurologie, F-54000 Nancy, France; 3 Université de Lorraine, CHRU-Nancy, Service de Neurochirurgie, F-54000 Nancy, France

**Keywords:** cortical face network, electrical brain stimulation, effective connectivity, frequency-tagging, SEEG

## Abstract

A major scientific objective of cognitive neuroscience is to define cortico-cortical functional connections supporting cognitive functions. Here, we use an original approach combining frequency-tagging and direct electrical stimulation (DES) to test for bidirectional and cross-hemispheric category-specific modulations within the human cortical face network. A unique patient bilaterally implanted with depth electrodes in multiple face-selective cortical regions of the ventral occipito-temporal cortex (VOTC) was shown 70 s sequences of variable natural object images at a 6 Hz rate, objectively identifying deviant face-selective neural activity at 1.2 Hz (i.e., every five images). Concurrent electrical stimulation was separately applied for 10 seconds on four independently defined face-selective sites in the right and left VOTC. Upon stimulation, we observed reduced or even abolished face-selective neural activity locally and, most interestingly, at distant VOTC recording sites. Remote DES effects were found up to the anterior temporal lobe (ATL) in both forward and backward directions along the VOTC, as well as across the two hemispheres. This reduction was specific to face-selective neural activity, with the general 6 Hz visual response being mostly unaffected. Overall, these results shed light on the functional connectivity of the cortical face-selective network, supporting its non-hierarchical organization as well as bidirectional effective category-selective connections between posterior ‘core’ regions and the ATL. They also pave the way for widespread and systematic development of this approach to better understand the functional and effective connectivity of human brain networks.

## 1. Introduction

A major objective of cognitive neuroscience research is to understand how large-scale networks of specialized cortical regions support cognitive functions. Human face recognition relies on a wide bilateral network of category-selective regions distributed in the occipital and temporal lobes [1,2,3,4,5,6]; this makes it an ideal model for characterizing the functional connectivity of the human brain. Specifically, face-selective brain regions (i.e., usually defined as regions in which populations of neurons respond more to faces than objects) have been consistently disclosed with functional magnetic resonance imaging (fMRI) in the lateral portion of the inferior occipital lobe (IOG, often labeled “occipital face area” or OFA [7]), the lateral portion of the posterior/middle fusiform gyrus (LatFG, often labeled “fusiform face area” or FFA [8]; see [9] in intracerebral recordings) with more recent proposals of several face-selective clusters in this region: FFA1 and FFA2 or pFus-faces and mFus-faces [10,11], as well as the posterior part of the superior temporal sulcus (pSTS) [12,13], all with a right hemispheric predominance. Face-selective clusters have also been found less systematically in the anterior temporal lobe (ATL; [14,15,16,17]; see also [9,18]).

The cortical face network has been deeply explored with neuroimaging, providing information about its structural connectivity (i.e., white matter tracts using Diffusion Tensor Imaging or DTI), functional connectivity (i.e., resting-state and task-state correlations of hemodynamic activity over time) and effective connectivity (i.e., the influence of brain regions over others using dynamic causal modeling, DCM, or more rarely, Granger Causality) [5,19,20,21,22,23,24,25,26,27]. Overall, these studies point to strong connections between the IOG/OFA and LatMidFG/FFA(s) but weak connections between these ventral occipito-temporal cortical (VOTC) regions and face-selective clusters of the (p)STS. This supports the view of two segregated neural face recognition systems: a dorsal system involved in the extraction of dynamic aspects of faces, such as facial expression and head or eye-gaze direction; and a ventral system in the VOTC that supports the recognition of relatively invariant aspects of faces, especially identity [2,3,6,28,29].

In line with the dominant view behind the neural organization of the visual recognition system [30,31], most theoretical models of human face recognition adopt a hierarchical organization of the category-selective network, with face-selective regions being recruited in succession along the postero-anterior axis, both along the VOTC and STS [2,3,4,28,32,33] (for this hierarchical view of STS face-selective clusters in non-human primates, see also [34,35,36]). Specifically, in the human VOTC, face-selective activity is thought to emerge in the OFA, feeding to the FFA(s) and then the ATL-faces regions (see above references). However, this hierarchical view has been questioned by lesion studies [25,37,38] and DCM [5,39] as well as DTI [5,22,24] studies, suggesting direct connections from early visual cortices to the FFA(s), i.e., bypassing the OFA, as well as bidirectional connections between the OFA and FFA.

Another important issue regarding the architecture of the ventral face-selective network concerns the connectivity between posterior regions (OFA and FFAs) and the ATL. Indeed, since ATL regions are hardly detected by fMRI studies due to magnetic susceptibility artifacts, little is known about the functional connectivity of the regions located anteriorly to the FFA [40]. Yet, intracranial EEG studies have recorded large face-selective activity across the ATL [9,18,41], with the region located just anteriorly to the FFA, i.e., the anterior fusiform gyrus (antFG), being particularly involved and critical for face identity recognition [40,42,43,44,45] (see direct electrical stimulations studies [46,47]).

Considering these elements altogether, the goal of the present study is to provide original information about the functional/effective connectivity of the human cortical face network, especially testing for bidirectional, cross-hemispheric and postero-anterior connections. To achieve this goal, we used a recently developed original approach that employs direct electrical stimulation (DES; [48,49,50,51]) to a targeted site through intracerebral electrodes, while concomitantly measuring frequency-tagged visually elicited neural activity across other brain regions [52]. Here, we apply this approach systematically to a unique case, patient YR, who benefited from a dense sampling of the bilateral VOTC, with intracerebral electrodes crossing multiple face-selective regions as defined independently.

## 2. Materials and Methods

### 2.1. Case Description

Subject YR was a right-handed 34-year-old man affected by refractory focal epilepsy. He underwent stereo-electroencephalography (SEEG) in March 2022 as part of the clinical investigation for his epilepsy. Following SEEG exploration, an independent epileptic focus was found in the right medial temporal lobe. Patient YR gave written consent for the experimental procedures that were administered during his SEEG exploration and that were part of the clinical investigation. Before the SEEG procedure, a series of behavioral tests were performed to specifically assess YR’s performance at face/object recognition (Benton Facial Recognition Test Electronic version, BFRT-c [53]; Face and car delayed matching [54]; Famous and non-famous face simultaneous matching [47]; Face memory [47]; Famous face and name pointing [47]). The results are shown in Table S1. YR’s face identity recognition ability was lower than that of matched control participants as assessed by the BFRT-c and matching tasks with pictures of unfamiliar and famous faces (performances compared by using the modified t-test of Crawford and Howell for single-case studies; [55]). However, he was also relatively impaired at matching pictures of cars, suggesting a non-specific impairment (see [56]).

### 2.2. Stereotactic Placement of Intracerebral Electrodes

**Figure 1:**
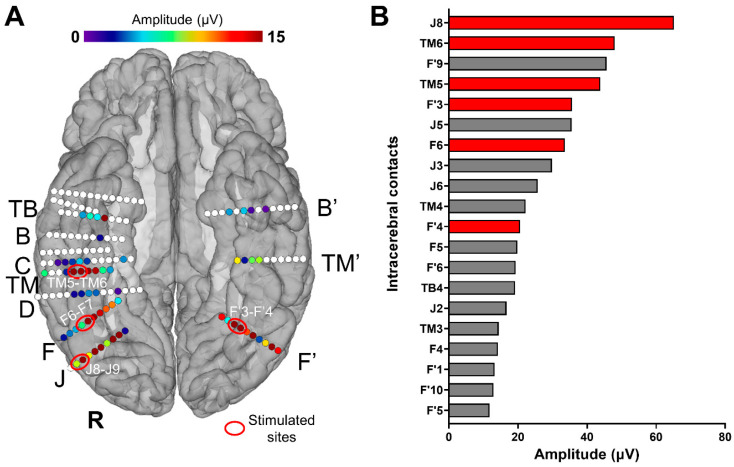
(**A**) Spatial distribution of face-selective responses (Face Categorization paradigm) displayed on a reconstructed cortical surface of subject YR’s brain. Each circle represents a single contact, colored circles correspond to face-selective contacts (*p* < 0.001; Z-score > 3.1, uncorrected) color-coded according to their face-selective response amplitude, and white-filled circles correspond to contacts that are not face-selective. The labels of some of the electrodes implanted in YR’s VOTC are indicated outside the brain (only the electrodes with at least one face-selective contact). The contacts that were selected for the stimulations are circled (in red) and labeled (e.g., TM5-TM6). (**B**) Top 20 (out of 142) VOTC intracerebral contacts with the highest amplitudes on the FPVS/SEEG paradigm Face Categorization (i.e., face-selective responses, low-frequency bands). Baseline-subtracted amplitudes are shown for the frequency of interest (1.2 Hz and harmonics). Note that all these contacts showed a significant face-selective response (Z-score > 3.1). The names of the contacts are indicated on the left; those in red are the contacts that were selected for the electrical stimulations during FPVS. For high-frequency bands, see Figure S1.

The patient was stereotactically implanted with intracerebral electrodes (Dixi Medical, Besançon, France) to delineate the seizure onset zone [57]. Each electrode consisted of a cylinder of 0.8 mm diameter and contained a linear array of 8–15 recording contacts, each 2 mm in length, separated by 1.5 mm from edge to edge. The sites of electrode implantation were determined based on non-invasive data collected during an earlier phase of the investigation. In total, 16 electrodes were implanted, with 13 targeting the left and right VOTC (3 in the left and 10 in the right; Figure 1A). A postoperative non-stereotaxic CT scan was carried out and fused with a T1-weighted MRI to determine the anatomical position of each electrode. The SEEG signal was recorded at a 512 Hz sampling rate, and the reference electrode used during data acquisition was a midline prefrontal scalp electrode (Fpz).

### 2.3. Face-Selective Responses outside Electrical Stimulation

**Figure 2:**
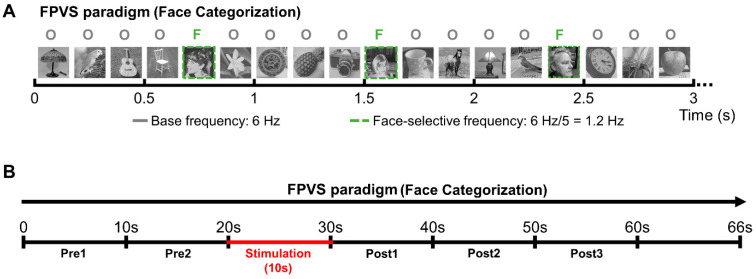
Experimental procedure. (**A**) The Face Categorization FPVS paradigm (from [55]) was administered either outside stimulation to independently define the most face-selective contacts or during the stimulation sessions to study the connectivity of the face-selective network. (**B**) Schematic representation of the stimulation sessions. Patient YR was presented with sequences of the Face Categorization paradigm; after about 20 s of visual presentation, intracerebral electrical stimulation was launched for 10 s. Portions of SEEG recordings corresponding to the FPVS sequence were then divided into periods of 10 s: 2 periods before stimulation (*Pre1* and *Pre2*), 1 stimulation period (*Stim*) and 3 periods after stimulation (*Post1*, *Post2*, and *Post3*).

To identify face-selective contacts, we used fast periodic visual stimulation (FPVS, or “frequency-tagging”), defined as the presentation of repeated stimuli at a fixed rate, i.e., periodic stimulation, that generates a periodic change in voltage amplitude in the recorded electrical activity, with a well-validated procedure (in scalp EEG: e.g., [58]; in SEEG, e.g., [9]; see [59] for review) (Figure 2A).

#### 2.3.1. Stimuli and Procedure

Two hundred grayscale natural images of various non-face objects (from 14 non-face categories: cats, dogs, horses, birds, flowers, fruits, vegetables, houseplants, phones, chairs, cameras, dishes, guitars, and lamps) and 50 grayscale natural images of faces were used (the same stimuli used in [9,58]). Subject YR viewed 3 continuous sequences of natural images of objects presented at a fast rate of 6 Hz. Images of faces appear periodically every 5th stimulus. This way, the neural activity that is common to faces and nonface stimuli is expressed at 6 Hz and harmonics (12 Hz, 18 Hz, etc.), while differential (i.e., selective) responses to faces are expressed at 1.2 Hz (i.e., 6 Hz/5) and harmonics (2.4 Hz, 3.6 Hz, etc.). This second frequency of interest, created by adding images of faces at a fixed interval, allows us to record pure face-selective responses in contrast to the general visual responses that are expressed at the base frequency (6 Hz) [58]. A stimulation sequence lasted 70 s: 66 s of stimulation (79 faces) at full contrast flanked by 2 s of fade-in and fade-out, where contrast gradually increased or decreased, respectively. During each sequence, YR was instructed to fixate on a small black cross, presented continuously at the center of the stimuli, and to press a button when it briefly (500 ms) changed color (black to red).

#### 2.3.2. Analysis of Intracerebral FPVS Responses

Analyses were carried out using the free software Letswave 5, with a similar procedure as in recent reports (e.g., [60,61]). Portions of recordings corresponding to sequences of FPVS presentation were first extracted using segments exceeding the actual visual presentation length (74 s segments, −2 s to +72 s) and then cropped to an integer number of cycles beginning after the 2 s fade-in and ending before the 2 s fade-out (i.e., ending up with segments of 66 s). These sequences, acquired with a scalp reference electrode (Fpz), were re-referenced to a bipolar montage (i.e., using as reference the signal recorded at the adjacent contact located laterally along the electrode). The four sequences of the experiment were averaged in the time domain to increase SNR, and a Fast Fourier Transform (FFT) was then applied to these averaged segments.

Face-selective activity significantly above noise level at the face frequency and its harmonics was determined as follows: (1) the FFT spectrum was cut into 50 bin segments centered at the face frequency and harmonics until the last harmonic before the 6 Hz base frequency (i.e., 1.2, 2.4, 3.6, 4.8 Hz; general visual responses); (2) the amplitude values of these FFT segments were summed; and (3) the summed FFT spectrum was then transformed into a Z-score, computed as the difference between the amplitude at each frequency bin and the mean amplitude of the corresponding 48 surrounding bins (25 bins on each side, i.e., 50 bins, excluding the first bin directly adjacent to the bin of interest, i.e., 48 bins) divided by the standard deviation of amplitudes in the corresponding 48 surrounding bins [60,61]. A contact was considered face-selective if the Z-score at the face frequency bin exceeded 3.1 (*p* < 0.001).

Baseline-corrected amplitudes were computed as the difference between the amplitude at each frequency bin and the mean amplitude of the corresponding 48 surrounding bins (25 bins on each side, i.e., 50 bins, but excluding the 2 bins directly adjacent to the bin of interest, i.e., 48 bins). The amplitude of face-selective responses for each contact was then quantified at each contact as the sum of harmonics from the first until the 13th harmonic (1.2 Hz until 16.8 Hz), excluding the 4th and 9th harmonics (6 Hz and 12 Hz) that coincided with the base frequency [9].

### 2.4. Intracerebral Electrical Stimulations during FPVS

#### 2.4.1. Procedure

Intracerebral electrical stimulations (application of electrical current through intracerebral electrodes) were applied between several pairs of contacts on electrode F (F6-F7; right LatFG; Talairach coordinates of the mean point between contacts: x: 45.41, y: −55.04, z: −12.03), TM (TM5-TM6; right AntFG; Talairach coordinates of the mean point between contacts: x: 48.16, y: −30.41, z: −20.25), J (J8-J9; right IOG; Talairach coordinates of the mean point between contacts: x: 48.44, y: −73.32, z: −11.18), and F’ (F’3-F’4; left LatFG; Talairach coordinates of the mean point between contacts: x: −28.98, y: −58.25, z: −10.41) during sequences of the Face Categorization paradigm. These stimulation sites were selected based on their location and large amplitude of face-selective activity outside of stimulation (Figure 1). Considering the contacts with the largest face-selective activity, we selected one stimulation site for each region of interest (e.g., right AntFG). Due to limited exploration time, the face-selective right AntCOS (close to the temporal pole, TB4) and left IOG (contacts F’6 to F’10) were not included in the selected stimulation sites (see Figure 1B).

During the stimulation sequences, in line with our standard procedure [52], patient YR was asked to fixate on the central cross without responding to color changes but to raise his hand if he perceived a change during the sequence, keeping his hand raised for the duration of the perceived effect. YR never reported any change of perception during stimulation. Subject YR was lying in his hospital bed positioned in front of the computer screen at approximately a 70 cm distance. A Face Categorization sequence was launched, running for 70 s (including 2 s of fade-in and 2 s of fade-out, so that the full-contrast sequence lasted 66 s); the 10 s bipolar stimulations (1.0 mA, biphasic square wave electrical pulses with 1050 µs width at 55 Hz, typical in parameters in SEEG; [62,63,64,65,66], see [51]) were manually triggered approximately 22 s after the onset of the sequence (i.e., at 20 s of full contrast). After that, the sequences ran for approximately 38 s (i.e., 36 s at full contrast); throughout testing, the patient was not aware of the stimulation onset and termination (Figure 2B).

Following this procedure, we performed 3 electrical stimulation sessions during the Face Categorization paradigm on each of the selected sites—F6-F7, TM5-TM6, J8-J9, and F’3-F’4 (i.e., 12 stimulations in total). No post-discharges were induced by these stimulations.

#### 2.4.2. Analysis of Intracerebral FPVS Responses before, during and after Stimulation

SEEG recordings obtained during the 66 s full-contrast sequences were divided into periods of 10 s (i.e., 2 periods before stimulation, 1 stimulation period, and 3 periods after stimulation; see Figure 2B). Given that the stimulation was triggered manually, the stimulation onset varied slightly across stimulation sessions; on average, the stimulation was administered after 21 s (21.33 ± 0.17) of full-contrast visual presentation.

These sequences were acquired with a scalp electrode (Fpz) as a reference channel and were then re-referenced to a bipolar montage, as described earlier for the non-stimulated sequences. FFT was applied to the 10 s segments, which were then averaged across stimulation sessions separately for each stimulation site and period (e.g., averaging all the *Pre1* segments relative to the stimulations on F6-F7). These averaged FFTs were then cropped into segments of 20 bins centered at the response frequencies (1.2 Hz) and harmonics, up until 10.8 Hz (i.e., including the first 8 harmonics, except the 4th harmonic, as it corresponds to the base frequency 6 Hz). The same was done for the base frequencies (6 Hz) and their harmonics (the first 3 harmonics, up to 24 Hz). The number of bins used here differs from the one mentioned above for the FPVS sequences without stimulation because of the lower frequency resolution that characterizes these recordings (i.e., 70 s vs. 10 s recordings). The number of harmonics included in the analysis was based on the highest number of consecutive harmonics that showed a significant response across contacts (Z-score > 3.1; i.e., *p* < 0.001).

The amplitude values of these FFT segments were then summed, and baseline-corrected amplitudes were obtained as the difference between the amplitude at each frequency bin and the mean amplitude of 18 corresponding surrounding bins (10 bins on each side, i.e., 20 bins, but excluding the 2 bins directly adjacent to the bin of interest, i.e., 18 bins).

#### 2.4.3. Statistical Analyses

In order to observe putative amplitude modulation of stimulation on the face-selective (1.2 Hz and harmonics) and general visual (6 Hz and harmonics) neural activity throughout the brain, we focused on the comparison between the stimulation period and the average of the two segments obtained before stimulation (*Pre1* and *Pre2*: *PreGA*).

The analysis of the FPVS sequences run during stimulation was limited to the face-selective contacts defined in the three non-stimulated sequences (*n* = 61; Figure 1A). To examine the amplitude modulation of the mean face-selective and general visual response at the single contact/region level, we computed the amplitude decrease for each contact by subtracting the FFT spectra of the stimulation period from the average of *Pre1* and *Pre2* (*PreGA* minus *Stim*); we then transformed the result into a Z-score (difference between the amplitude at each frequency bin and the mean amplitude of the corresponding 18 surrounding bins divided by the standard deviation of amplitudes of these 18 bins). A contact was considered as showing a significant amplitude reduction of the face-selective or general visual response during stimulation if the Z-score exceeded 2.32 (*p* < 0.01).

Next, we performed a global amplitude analysis to observe the amplitude modulation of the mean face-selective and general visual response across the pool of contacts throughout the sequences (contacts with significant face-selective responses outside of stimulation, excluding the stimulated contacts). The baseline-corrected amplitudes of these contacts were averaged separately for the face-selective and general visual responses for each period and compared to those obtained during non-stimulated sequences. The 3 non-stimulated sequences we recorded outside of stimulation were processed the same way as the stimulated sequences. We statistically compared the difference between the average of *Pre1* and *Pre2* and the stimulation periods (*PreGA-Stim*) for the stimulated sequences and the difference between the corresponding periods for the non-stimulated sequences (amplitude of the third period subtracted from the average of the first and second period, i.e., average of *P1* and *P2* minus *P3*) using a two-tailed paired permutation test (Nperm: 40,000).

## 3. Results

### 3.1. Summary of the Experimental Plan

We identified six main face-selective regions based on the amplitude of their face-selective responses (Face Categorization paradigm) and their anatomical localization (Figure 1): right IOG (contacts J6 to J9), right LatFG (F3 to F6 and J2 to J5), right AntFG (TM 2 to TM 6), right AntCOS close to the temporal pole (TB4), left IOG (contacts F’6 to F’10) and left LatFG (F’3 to F’5). To study the effective connectivity of the cortical face system, we selected four different sites, i.e., pairs of contacts (Figure 1): J8-J9 in the right IOG, F6-F7 in the right LatFG, TM5-TM6 in the right AntFG, and F’3-F’4 in the left LatFG (the two remaining selective regions were not included due to time constraints). Note that the amplitude quantification of face-selective responses in the high-frequency bands (30–160 Hz) yields a very similar ranking (except for contacts F6-F7, which appear less face-selective; see Figure S1) and that the overall significant contacts were reduced (25 versus 61; in line with [41]). These four pairs of contacts were then electrically stimulated for 10 s while the subject was presented with the FPVS Face Categorization paradigm. The combined use of FPVS, providing objective high SNR responses, and of DES allows us to assess the local (i.e., within the same anatomical region of the stimulated site) and remote neural effects of electrical stimulation on specific regions of the cortical face network by recording in real time the FPVS face-selective response before, during, and after stimulation throughout the brain [52]. The large number of electrodes implanted in YR’s bilateral VOTC allowed us to explore a large portion of the cortical face network. Out of the 142 contacts recorded in the bilateral VOTC, 61 were face-selective according to the FPVS Face Categorization paradigm. These contacts were distributed over the VOTC bilaterally (Z-score > 3.1, *p* < 0.001, Figure 1A).

Subject YR was shown sequences that lasted 66 s in full contrast. After 20 s, a bipolar stimulation of one of the of the four selected regions was administered for 10 s. During the whole sequence, the neurophysiological activity of all the implanted regions was recorded. SEEG recordings corresponding to the FPVS sequences were divided into periods of 10 s for analyses (Figure 2B; two periods before stimulation, *Pre1* and *Pre2*; one stimulation period; and three periods after stimulation, *Post1*, *Post2*, and *Post3*).

### 3.2. Stimulating Nodes of the Cortical Face Network Induces a Reduction of Face-Selective Neural Activity across the VOTC

**Figure 3:**
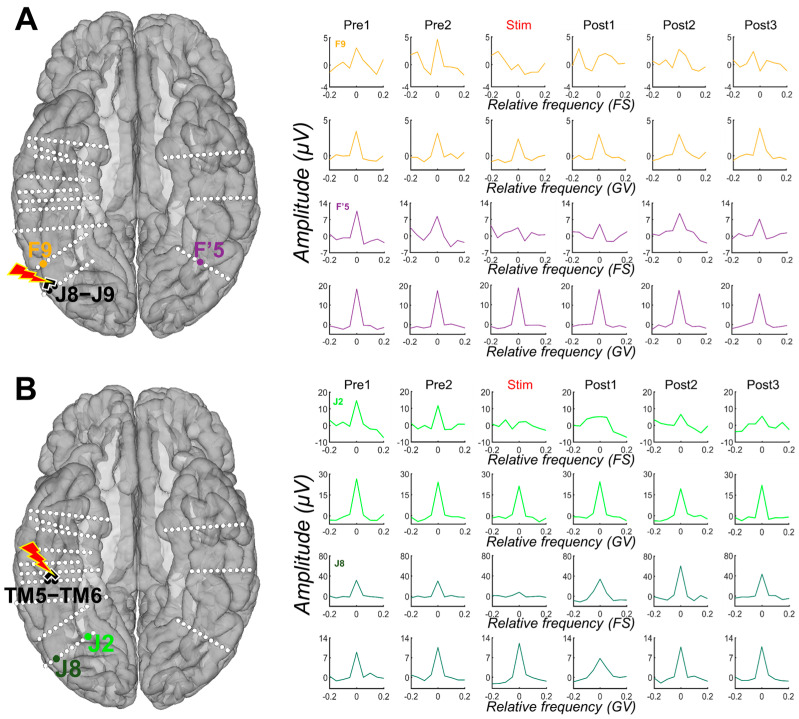
Examples of amplitude variation for the face-selective and general visual responses throughout the Face Categorization FPVS sequences with electrical stimulation of the right AntFG (TM5-TM6) and right IOG (J8-J9). Mean baseline-corrected FFT of the face-selective (FS) and general visual (GV) responses across 3 stimulation sessions of contacts TM5-TM6 (see (**B**)) and J8-J9 (see (**A**)) are shown for each period, before stimulation (*Pre1*, *Pre2*), during stimulation, and after (*Post1*, *Post2*, *Post3*). These remote contacts showed a decrease, or even suppression, of the face-selective activity relative to the stimulation, while the general visual response remained unaffected. For more examples, see Figure S2.

After quantifying the face-selective and general visual responses in each period (*Pre1*, *Pre2*, *Stim*, *Post1*, *Post2*, and *Post3*) for each contact, we observed that stimulation of the four face-selective sites (F6-F7, J8-J9, TM5-TM6, and F’3-F4) induced strong decreases of the face-selective activity not only in the stimulated region but also remotely (Figure 3). This effect was specific to the stimulation period and sometimes remained during the post-stimulation periods, with a gradual return to the pre-stimulation amplitude (Figure 3). Strikingly, this effect appeared to be restricted to the face-selective response for most of the contacts, with no obvious decreases observed for the general visual response (Figure 3; see also Figure S2).

**Figure 4:**
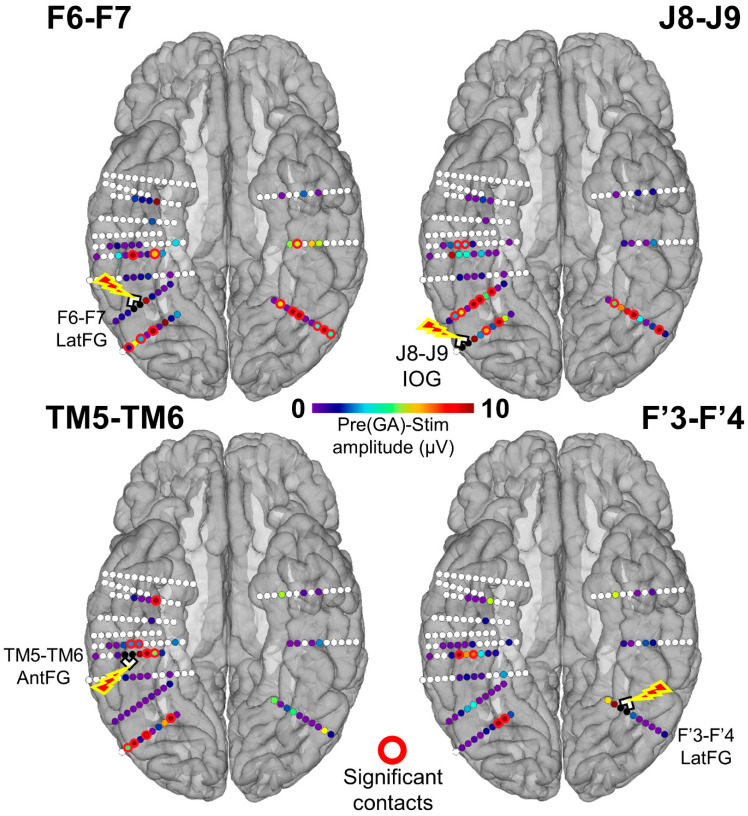
Spatial distribution of the face-selective response amplitude decrease during stimulation for each stimulated site displayed on a reconstructed cortical surface of subject YR’s brain. Contacts of interest (pool of face-selective contacts outside of stimulation) are color-coded according to the baseline-corrected amplitude difference between the average of *Pre1* and *Pre2* and the stimulation periods (stimulation effect). Contacts with a significant difference are circled in red (Z-score > 2.32, *p* < 0.01). See Figure S3 for a similar observation of the general visual response amplitude decrease during stimulation for each stimulated site.

**Figure 5:**
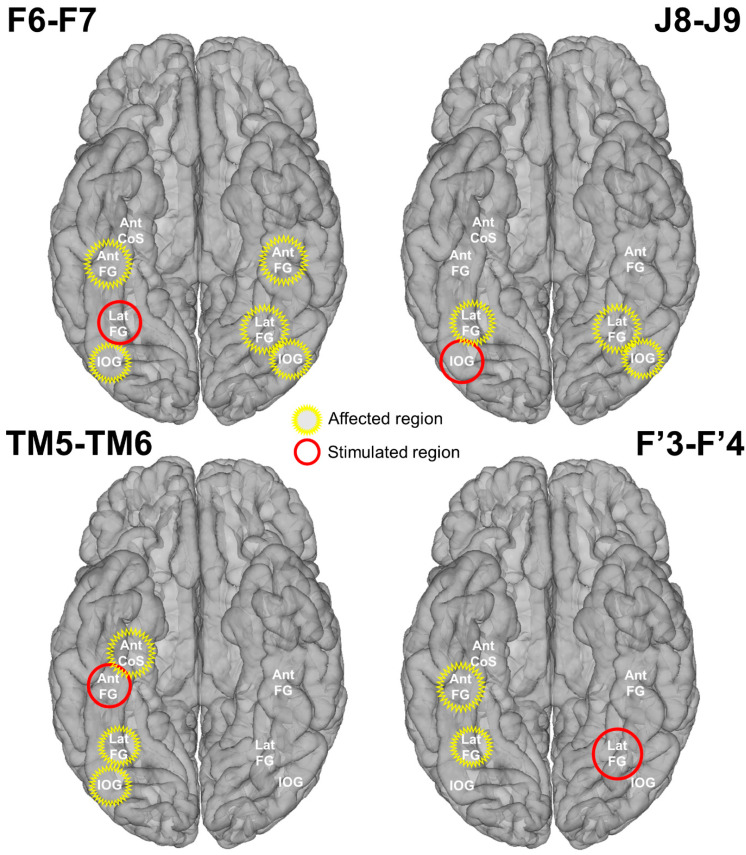
Schematic representation of the stimulation effect on main face-selective regions. The stimulated and affected regions are indicated in red and yellow, respectively. A region was considered affected if at least one contact showed a significant decrease of the face-selective response due to stimulation.

We first quantified the number of individual contacts showing a significant amplitude decrease of the face-selective response relative to the stimulation by subtracting the baseline-corrected amplitude computed during stimulation from the average of the amplitudes computed during the *Pre1* and *Pre2* periods (*PreGA-Stim*) for each contact included in the face-selective pool (Figure 2A). A contact was considered as showing a significant face-selective amplitude reduction during stimulation if the Z-score exceeded 2.32 (*p* < 0.01). We restricted this analysis to the 61 contacts that showed significant face-selective responses (FPVS Face Categorization paradigm) outside of stimulation. While stimulating the right IOG (J8-J9), 10 contacts in the bilateral LatFG and left IOG showed a significant reduction of the face-selective response. Upon right LatFG (F6-F7) stimulation, 12 contacts in the bilateral AntFG, left LatFG, and bilateral IOG showed a significant reduction of face-selectivity. While stimulating the left LatFG, four contacts in the right AntFG and LatFG showed a significant reduction of face-selectivity. Finally, while stimulating the right AntFG, eight contacts in the right AntCOS, IOG, and LatFG showed a significant reduction of face-selectivity. There was no statistical difference across regions in terms of the proportion of contacts with a significant decrease (*p* > 0.05; Fisher exact test). These results are shown in Figure 4 and summarized at the level of anatomical regions in Figure 5.

Considering that we were able to electrically stimulate the LatFG in both hemispheres, we decided to directly compare left and right hemispheric stimulations. When stimulating the right LatFG, 39% of the left face-selective contacts (7/18) showed a significant decrease, while only 9% of right face-selective contacts (4/43) were affected when stimulating the left LatFG. This difference was statistically significant (*p* = 0.01; Fisher’s test). At the level of anatomical regions, the right LatFG affected five face-selective regions, while the left LatFG only affected two regions (Figure 5).

**Figure 6:**
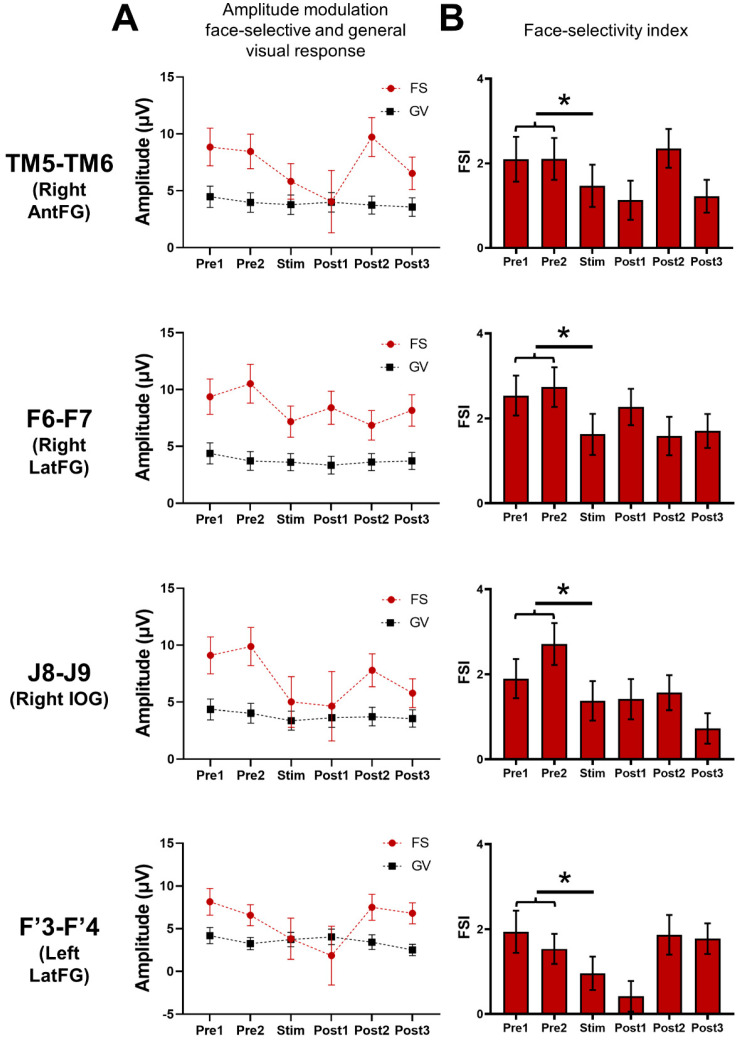
Global (i.e., across all significant contacts) variation of the baseline-corrected amplitudes of the face-selective (FS) and general visual (GV) responses and the face-selectivity index observed throughout the FPVS sequences. (**A**) Mean baseline-corrected amplitudes of the face-selective and general visual responses throughout the FPVS sequence across the pool of contacts of interest (i.e., showing a significant face-selective response already outside stimulation, *n* = 61 minus the 2 stimulated contacts) during the stimulated (Stim F6-F7, J8-J9, TM5-TM6, F’3-F’4) sequences. (**B**) This face-selective index (FSI) was calculated by subtracting the GV amplitude (i.e., 6 Hz and 3 harmonics) from the face-selective amplitude (i.e., 1.2 Hz and 7 harmonics) for each period (FS-GV); this difference was transformed into a Z-score. We then compared the FSI modulation computed for the average of *Pre1* and *Pre2* and the FSI computed during the stimulation (*Stim*). (*) indicates a significant difference at *p* < 0.05 (two-tailed paired *t*-tests).

Next, we quantified the overall amplitude modulation of the face-selective response across the pool of non-stimulated contacts, whether they showed a reduction during stimulation or not (61 face-selective contacts minus the 2 respective stimulated contacts, i.e., 59) for each stimulation site (Figure 6A). For each site, we observed a decrease of the face-selective response during stimulation. To statistically assess this reduction, we compared these stimulation sessions to the variation of responses observed during sequences acquired outside stimulation (subtracting the baseline-corrected amplitude computed during stimulation from the average of the amplitudes computed during the *Pre1* and *Pre2* periods for stimulated sequences versus third minus the average of the first and second periods for the non-stimulated sequences). Importantly, the face-selective responses remained highly stable across periods for the non-stimulated sequences (Figure S4). The face-selective amplitude decrease was significantly higher for the stimulated sequences compared to the non-stimulated sequences for all the modulated sites (F6-F7: *p* < 0.0001; J8-J9: *p* < 0001; TM5-TM6: *p* = 0.02; F’3-F’4: *p* = 0.02; two-tailed paired permutation tests; see Figure S4). We performed the same analysis to compare the effect of the right and left LatFG. To do so, we compared the amplitude effect of left and right LatFG, restricting our pool of contacts to those located in the other hemisphere (i.e., on 18 face-selective contacts in the left hemisphere for F6-F7 and 46 face-selective contacts in the right hemisphere for F’3-F’4; Figure S5). When compared to non-stimulated sequences, we observed a decrease for the stimulated sequences for the right LatFG only, although at the limit of significance (F6-F7: *p* = 0.051; F’3-F’4: *p* = 0.11; two-tailed paired permutation tests).

### 3.3. Predominant Reduction of the Face-Selective Response Relative to Common Neural Activity to Faces and Objects

As shown in Figure 3, some contacts showed a reduction of the face-selective response with either no or less concomitant decrease for the general visual response. We quantified the number of individual contacts showing a significant amplitude decrease of the general visual response relative to the stimulation for each contact included in the pool of contacts (61), as we did for the face-selective response (*PreGA-Stim*). Almost no contact with a significant decrease of the face-selective response concomitantly showed a reduction of the general visual response (Table S2; the number of contacts remains very low with a more liberal statistical threshold, Table S3).

We also quantified the overall amplitude modulation of the general visual response across the pool of non-stimulated contacts (i.e., 61 minus the stimulated sites, 59 contacts; Figure 6A). The stimulation did not specifically change the general visual amplitude. The stimulated and non-stimulated sessions were compared in the same way as for the face-selective response (Figure S4). While there was a significant reduction for the face-selective response for each site, no such reduction was observed for the general visual response (F6-F7 *p* = 0.86; J8-J9 *p* = 0.08; TM5-TM6 *p* = 0.99; F’3-F’4: *p* = 0.64; two-tailed paired permutation tests).

Finally, we computed a face-selectivity index (FSI) by subtracting the general visual amplitude (i.e., 6 Hz and three harmonics) from the face-selective amplitude (i.e., 1.2 Hz and seven harmonics) for each period; this difference was then transformed into a Z-score (difference between the amplitude at each frequency bin and the mean amplitude of the corresponding 18 surrounding bins, divided by the standard deviation of amplitudes of these 18 bins) (Figure 6B). This FSI provides an estimation of the magnitude of the face-selective response relative to the overall visual responsiveness of the contact. The FSI was significantly reduced during the stimulation period compared to the pre-stimulation periods for each site (TM5-TM6: *p* = 0.004; F6-F7: *p* = 0.0000003; J8-J9: *p* = 0.000009; F’3-F’4: *p* = 0.001; two-tailed paired *t*-test), showing that the stimulation disproportionally reduced the face-selective response compared to the general visual response.

### 3.4. Functional Specificity of the Stimulation Effects

**Figure 7:**
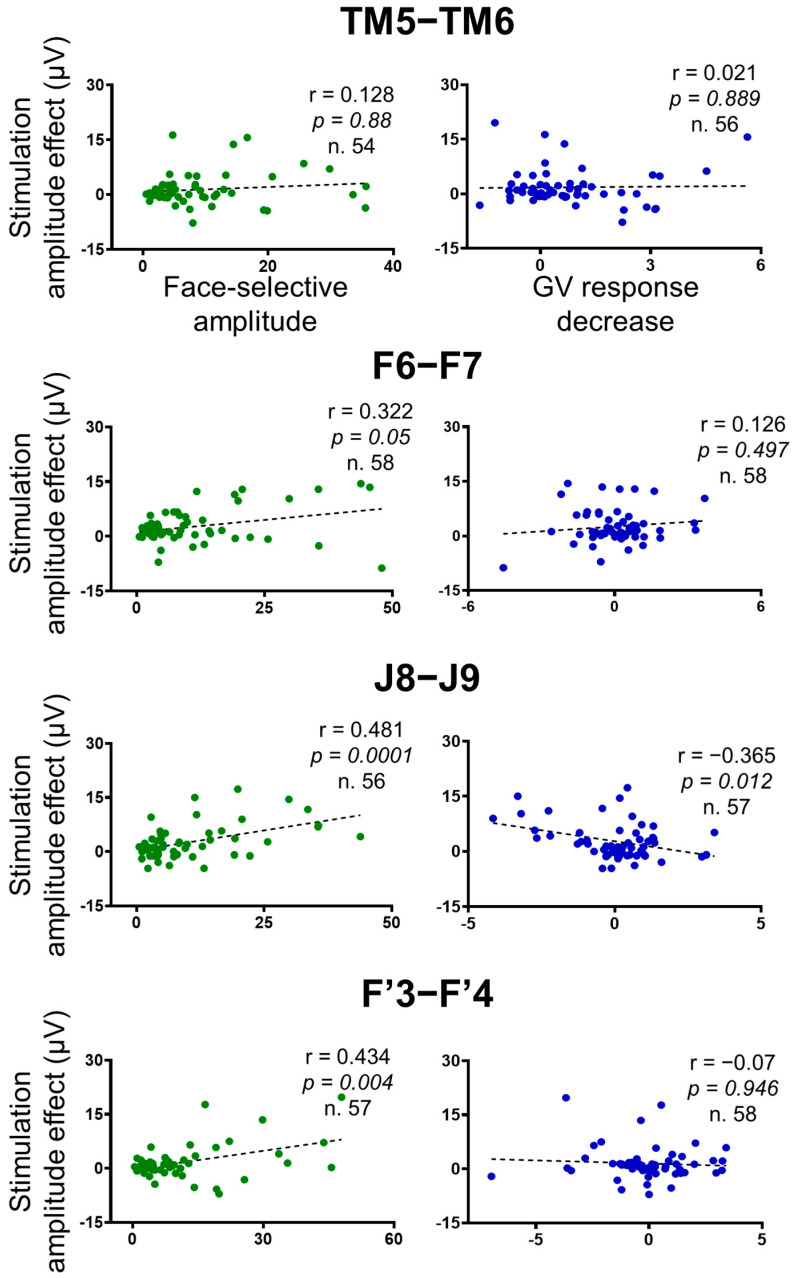
Correlation plots between the stimulation amplitude effect on the face-selective responses (baseline-corrected amplitude difference between the average of *Pre1* and *Pre2* and stimulation periods), independent face-selective responses (computed outside stimulation), and the stimulation amplitude effect on the general visual responses across the contacts of interest (*n* = 61 minus the 2 stimulated contacts). Outliers (Z-score>3) were removed. The Pearson correlation coefficient, the *p*-values, and the number of contacts included in the analyses are indicated for each correlation. False Discovery Rate (FDR) corrections were applied to control for multiple comparisons [67].

To evaluate to what extent the stimulation specifically affects the cortical face network, we computed correlations between the stimulation effect on the face-selective amplitude (*PreGA-Stim*) and face-selective responses acquired outside the stimulation sessions, the stimulation effect on the general visual response, as well as physical measurements (Euclidean distance from the stimulation site and the amplitude of the stimulation artifact), across the 59 corresponding non-stimulated contacts (Figure 7). All correlations were computed by removing outliers (Z-score > 3), and False Discovery Rate (FDR) corrections were applied to control for multiple comparisons [67].

We found highly significant positive correlations between the amplitude effect of the stimulation and the face-selective amplitude computed outside of stimulation for the F6-F7, F’3-F’4 and J8-J9 stimulations (Pearson correlations; F6-F7: r(56) = 0.322, *p* = 0.05; J8-J9: r(54) = 0.481, *p* = 0.0001; F’3-F’4: r(55) = 0.434, *p* = 0.004), showing that the more a contact was face-selective, the more the face-selective responses decreased during stimulation (Figure 7). However, no correlation was found for the TM5-TM6 stimulations (r(52) = 0.128, *p* = 0.88).

There was no significant positive correlation between the stimulation effect on the face-selective and the general visual responses for any of the stimulation sites (F6-F7: r(56) = 0.126, *p* = 0.497; J8-J9: r(55) = −0.365, *p* = 0.012; TM5-TM6: r(54) = 0.021, *p* = 0.889; F’3-F’4: r(56) = 0.07, *p* = 0.946) (Figure 7).

Lastly, there was no significant positive correlation for any of the stimulation sites when correlating the amplitude decrease of the face-selective response with the Euclidean distance from the stimulation site (F6-F7: r(57) = 0.09, *p* = 0.49; J8-J9: r(56) = −0.203, *p* = 0.14; TM5-TM6: r(55) = 0.025, *p* = 0.89; F’3-F’4: r(56) = 0.041, *p* = 0.95) and the amplitude of the stimulation artifact (F6-F7: r(55) = −0.111, *p* = 0.47; J8-J9: r(56) = 0.176, *p* = 0.185; TM5-TM6: r(54) = 0.139, *p* = 0.82; F’3-F’4: r(56) = 0.009, *p* = 0.95) (see Figure S6).

## 4. Discussion

Using an original combination of concurrent direct electrical stimulation and visual frequency-tagging [52], we causally modulated face-selective neural activity in the VOTC of a unique case implanted with multiple depth electrodes in the cortical face network. We electrically stimulated four different face-selective regions (right AntFG, right and left latFG, and right IOG) and observed a significant decrease of face-selectivity in remote face-selective regions, thus fully supporting the view that the effects of intracerebral direct electrical stimulation extend well beyond the stimulation site [48,49,51]. Overall, we show that (1) each stimulation site affected several remote face-selective contacts within and across hemispheres; (2) the stimulation effect was bidirectional along the postero-anterior axis; and (3) the ATL, a generally neglected region in the cortical face network, was affected by the stimulation of more posterior regions, while, in turn, DES to this region also reduced face-selective activity in these regions. Before discussing the significance of these results, especially in light of current theoretical models of human face recognition, we take a few brief considerations into account regarding the present methodology, the nature of our general observations, and their potential limitations.

Firstly, thanks to the frequency-tagging approach (see [68,69]), face-selective neural activity can be objectively identified and quantified throughout the stimulation procedure without being affected by stimulation artifacts (i.e., face-selective activity falling in small frequency bins at 1.2 Hz and harmonics, with stimulation artifacts being recorded at 55 Hz and harmonics; see [52]). This is a major strength of the present study. Secondly, compared to our recent report demonstrating the validity of this approach in another case [52], here we demonstrate DES effects on face-selective neural activity. In fact, and strikingly, DES disrupted or abolished face-selective activity while sparing general visual responses (at 6 Hz and harmonics) even at the very same electrode contacts where both functional responses were recorded (e.g., Figure 3). Finally, and in line with the previous point, the correlation between the face-selective amplitude decrease caused by DES and measures of face-selectivity acquired independently shows that electrical stimulation specifically affected the cortical face network.

Concerning the limitations, we identify three main points. First, the original evidence reported here is based on a single case and must be strengthened by additional cases and, ideally, group studies in the future. However, given the specificity in terms of the number of implanted electrodes and their localization in each individual patient, and this implantation being directed strictly based on clinical criteria, such datasets may take years to collect and will always be difficult to combine meaningfully in group analyses. Here, the reported case has a particularly rare, dense implantation in several (VOTC) bilateral regions of the cortical face network, making it uniquely relevant for the purpose of this research. Moreover, we were able to perform a sufficiently large number of stimulation trials associated with clear effects to provide statistically significant effects, even for face-selective activity (unlike the recent first case in which the present methodology was validated; [52]). Second, the subject suffers from long-term epilepsy refractory to medication affecting his temporal lobe, raising the issue of a potential (partial) reorganization of grey matter function and/or cortical connectivity that may have affected our results (e.g., [70,71,72,73]). Yet, previous studies by our group, with FPVS in particular, have systematically demonstrated typical responses in such cases (e.g., remarkably congruent with fMRI in neurotypical individuals in terms of lateralization and localization of category-selective activity in the VOTC; see [9,60]; discussion in [59]). As a matter of fact, face-selectivity was found in all relevant regions of the cortical face network in this patient. Finally, despite the strong effects of DES on local and remote neural activity, patient YR did not experience any behavioral impairment or subjective changes of the perceived faces, contrary to the previous case. However, this could be attributed to a few reasons. First, the subject’s ability to recognize facial identity is below the normal range. Second, (unfamiliar) faces appear only briefly and relatively rarely, i.e., every five non-face objects, in the paradigm used here.

### 4.1. Evidence for Bidirectional Effective Connectivity between IOG/OFA and LatFG/FFA

Inspired by the general view of the human visual recognition system, most neuro-functional models of the cortical face network are hierarchically organized, with increasingly complex representations thought to be built step by step, from posterior to anterior brain regions (e.g., [2,4,19,28,32,33,74]). This hierarchical processing mode has been particularly advocated for face-selective activity in the LatFG/FFA, which is thought to follow and build upon neural activity in the posteriorly located IOG/OFA [2,19,28,33,75].

However, thanks originally to lesion studies in single cases ([37,38], see also [25]), then time-resolved fMRI studies [76], intracranial recordings [77] and more recently, DTI studies [24], it is now widely believed that direct connections from early visual cortices to the LatFG/FFA can lead to face-selective activity in this region, bypassing the IOG/OFA [78,79].

Given the prevalence of bidirectional anatomical connections between cortical areas of the visual system and beyond [80,81,82,83], most models of human face recognition have also implied/incorporated such bidirectional connections between cortical face-selective areas such as the OFA and FFA, as also supported by (dynamic causal) modeling studies of fMRI signals ([5,21,27], but see [19,23]). However, to our knowledge, direct evidence of effective bidirectional connectivity between these two key regions of the cortical face network had never been demonstrated, let alone in the antero-posterior direction (i.e., FFA to OFA). Here, since stimulation of the LatFG decreased face-selectivity in the IOG and vice versa, the present results, albeit collected in a single case, provide strong evidence for such effective, bidirectional connectivity in the right hemisphere between the face-selective IOG (“OFA”) and latFG (“FFA”). While we cannot fully exclude that effective connectivity between these regions is mediated by other regions of the network (i.e., indirect), our results converge with indirect anatomical and functional evidence [5,24] that suggest direct reentrant (i.e., functional; [82]) connections between these critical regions of the cortical face network. Further studies are needed to test whether these proposed OFA-FFA bidirectional connections are also present in the left hemisphere and, most importantly, whether they critically support the elaboration of a full-face identity percept in the VOTC, as previously hypothesized [59,78,84]. This latter proposal could potentially be addressed through DES concurrently applied with frequency-tagging measures of face identity recognition [61], eliciting transient impairment in this function ([85,86]; see [51]).

### 4.2. Effective Connectivity of Face-Selective Anterior Temporal Lobe Regions

As mentioned above, very little is known about the functional connectivity of ATL face-selective regions, partly because of a strong fMRI signal drop-out due to a susceptibility artifact [87,88,89,90] that limits the recording of genuine fMRI face-selective responses and functional connectivity measures in this region (see, e.g., [40]). Yet, a handful of fMRI studies reported relatively weak face-selective activations close to the temporal pole (named either ATFP, ATL-faces or ATL-FA), resulting in a gap in face-selectivity between the most anterior portion of the FFA (sometimes called FFA2 or mFus-faces; [4]) and very anterior face-selective regions close to the temporal pole and corresponding the AntCOS region here [14,17,40,91]. Anatomically, the region where the artifact is the largest corresponds to the antFG. In contrast to fMRI, intracranial EEG studies reported face-selective activity all along the VOTC and ATL without any gap [59]. Moreover, among ATL regions, the right antFG records the largest face-selective response [9,41] and appears to be critical for FIR [46,47].

Here, stimulating the right middle LatFG decreased face-selectivity in the ipsilateral antFG and vice versa. Stimulation of the right antFG also affected face-selectivity in the ipsilateral antCOS and IOG. Altogether, these observations reveal a complex connectivity pattern, with connections between the two ATL face-selective regions (forward, at least because the antCOS was not stimulated), bidirectional connections between the right antFG and latFG, and backward connections from the antFG to IOG (see [92] for a functional connectivity study between the anatomically defined antFG and OFA/FFA). Despite large inter-species differences in cortical organization of face recognition between humans and macaque monkeys [93,94], these findings concur with effective connections from anterior to posterior face-selective regions observed along the monkey superior temporal sulcus, combining MRI and electrical microsimulation ([35]; see also [95] for similar results with direct injections of retrograde tracers). In humans, these backward connections along the (right) ventral cortical face network (IOG/latFG, latFG/antFG, perhaps antFG/antCOS) may play a role in shaping the category and identity selectivities of neuronal populations in posterior regions through multimodal semantic representations located in the ATL (see [40,79]).

### 4.3. Interhemispheric Effective Connectivity

Most connectivity studies, and consequently cortical models of human face recognition, have focused on intra-hemispheric connections between face-selective regions (DTI: [20,22]; TMS and fMRI: [33]; DCM: [19,23,27]; functional connectivity: [96]; but see [97]). Here, we show strong interhemispheric effective connectivity (i.e., effect on the left when stimulating the right hemisphere and vice versa). Contralateral effects concerned both corresponding and non-corresponding regions, and they were particularly strong for all stimulated regions, except perhaps for the right antFG. This result is in accordance with an fMRI functional connectivity study that highlights the importance of interhemispheric connections within the face-selective network by showing that correlations are greater between corresponding face regions in different hemispheres than between different face regions in the same hemisphere [97,98]. Despite the obvious limitations of a single case study, we attempted to assess the weight of these interhemispheric connections across both hemispheres. We found that the right latFG affected more contralateral contacts than the corresponding regions in the left, consistently with the well-known right hemispheric dominance for face recognition. In the future, these findings should be strengthened by additional recordings in larger samples.

## Data Availability

The data presented in this study are available on request from the corresponding author. The data are not publicly available due to privacy restrictions.

## Supplementary Materials

The following supporting information can be downloaded at: https://www.mdpi.com/article/10.3390/brainsci14090906/s1. Figure S1. Top 27 (out of 142) VOTC intracerebral contacts with the highest amplitudes on the FPVS/SEEG paradigm Face Categorization (i.e., face-selective responses) in the high-frequency bands. Figure S2. Other examples of amplitude variation for the face-selective (oddball frequency) and general visual (base frequency) responses throughout the Face Categorization FPVS sequences with electrical stimulation of the right (F6-F7) and left LatFG (J8-J9). Figure S3. Spatial distribution of the general visual response amplitude decrease during stimulation for each stimulated site. Figure S4. Global variation of the baseline-corrected amplitudes of the face-selective and general visual response observed throughout the FPVS sequences. Figure S5. Comparison of the effect evoked by the stimulation of the right and left LatFG throughout the FPVS sequences. Figure S6. Correlation plots between the stimulation amplitude effect for the face-selective responses (baseline-corrected amplitude difference between the average of *Pre1* and *Pre2* and stimulation periods) and physical measurements (Euclidean distance from the stimulation site and the amplitude of the stimulation artifact) across the contacts of interest. Table S1. Behavioral performance of YR and 5 control subjects in face/object recognition tasks. Table S2. Quantification of the stimulation effect for the face-selective and general visual responses (threshold Z-score > 2.32). Table S3. Quantification of the stimulation effect for the face-selective and general visual responses (threshold Z-score > 1.64).
