# Supplementary material for: Bidirectional and Cross-Hemispheric Modulations of Face-Selective Neural Activity Induced by Electrical Stimulation within the Human Cortical Face Network
Source: Brain Sci. 2024 Sep 6;14(9):906. doi: 10.3390/brainsci14090906 (PMC11429542; doi:10.3390/brainsci14090906)
Supplement: Supplementary file 1 [file brainsci-14-00906-s001.zip › brainsci-3140150-supplementary.pdf]

Supplementary materials

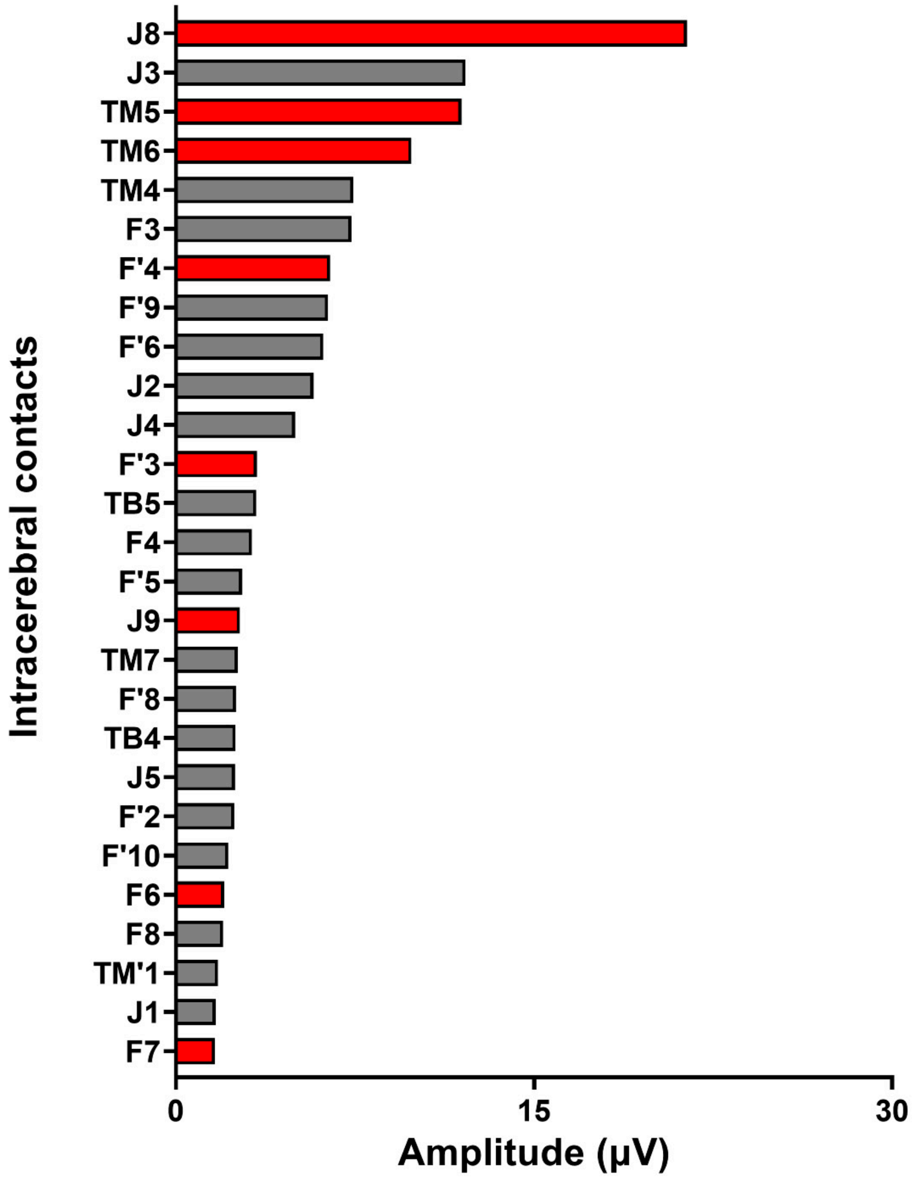

Figure S1. Top 27 (out of 142) VOTC intracerebral contacts with the highest amplitudes on the FPVS/SEEG paradigm Face Categorization (i.e., face-selective responses) in the high-frequency bands. *We examined the face-selective response in high-frequency broadband activity (i.e., between 30 and 160 Hz, "gamma activity"). Event-related spectral perturbations (ERSP) were computed using*

Letswave 5, similarly to what was reported in [41]. Variation in signal amplitude as a function of time and frequency was estimated by a Morlet wavelet transform on each SEEG segment from frequencies of 1 to 160 Hz, in 2 Hz increments. The number of cycles (i.e., central frequency) of the wavelet was adapted as a function of frequency from 2 cycles at the lowest frequency to 9 cycles at the highest frequency. The wavelet transform was computed on each time-sample and the resulting amplitude envelope was downsampled by a factor of 12 (i.e., to a 166.6 Hz sampling rate). Amplitude was normalized across time and frequency to obtain the percentage of power change generated by the stimulus onset relative to the mean power in a pre-stimulus time window (-1600 ms to -300 ms relative to the onset of the stimulation sequence). Then, the amplitude was averaged across frequencies (between 30 Hz and 160 Hz), the high-frequency broadband envelopes corresponding to the same condition were averaged in the time domain, and the frequency content of the high-frequency broadband envelope was transformed using a Fast Fourier transform. Significant responses in the high-frequency bands were detected similarly to the low-frequency bands. The quantification of amplitudes was done similarly as for responses in the low-frequency bands, i.e., summing amplitudes from the first until the 3rd harmonic (i.e., 1.2Hz until 4.8Hz). Note that all these contacts showed a significant face-selective response (z-score > 3.09) The names of the contacts are indicated on the left; in red are the contacts that were selected for the electrical stimulations during FPVS.

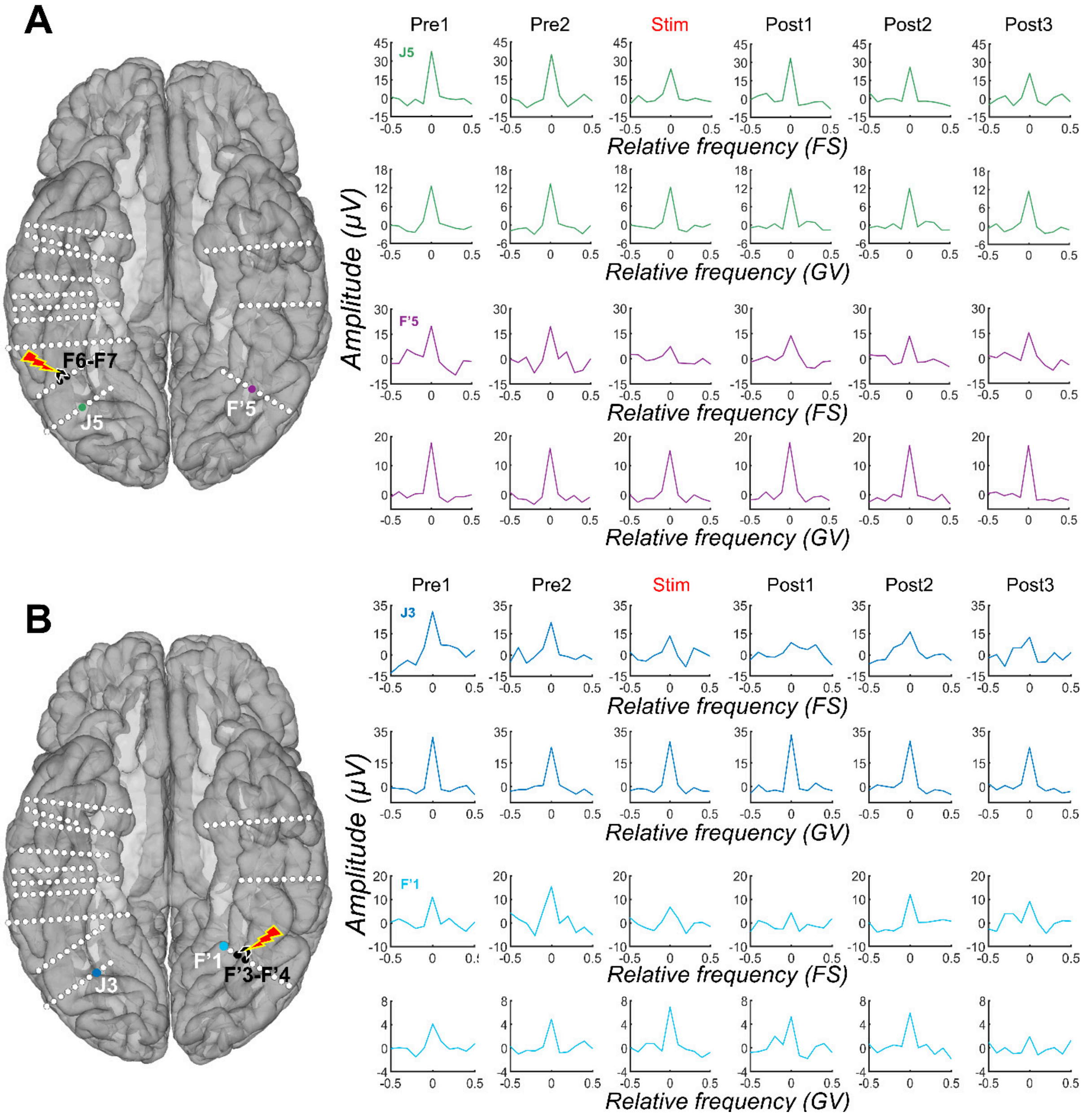

Figure S2. Other examples of amplitude variation for the face-selective (FS) and general visual (GV) responses throughout the Face Categorization FPVS sequences with electrical stimulation of the right (F6-F7) and left LatFG (F'3-F'4). *Mean baseline-corrected FFT of the FS and GV responses across 3 stimulation sessions of contacts F6-F7 (see **A**) and F'3-F'4 (see **B**) are shown for each period, before stimulation (Pre1, Pre2), during stimulation, and after (Post1, Post2, Post3). These remote contacts showed a decrease of the face-selective activity relative to the stimulation, while the general visual response remained unaffected.*

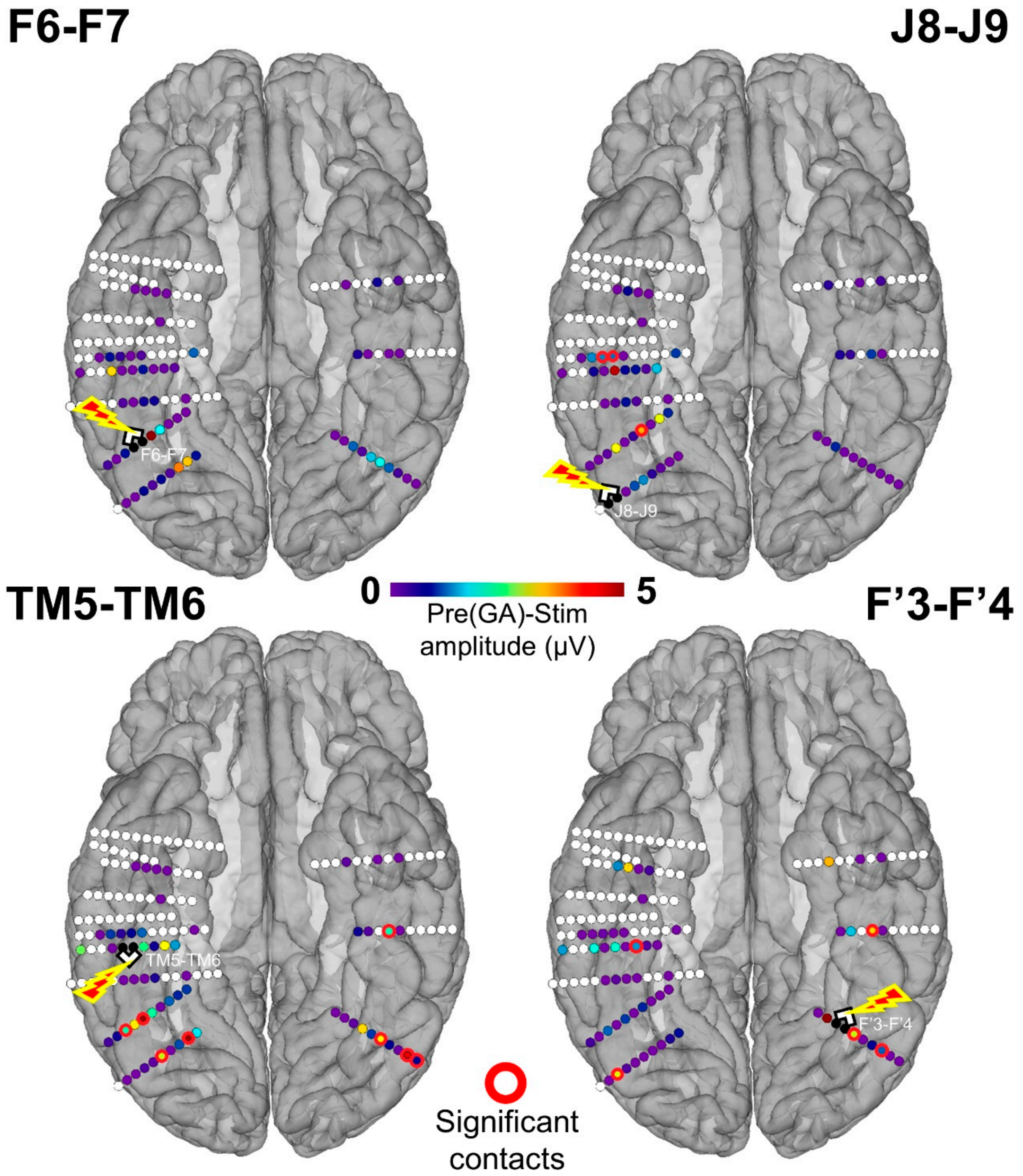

Figure S3. Spatial distribution of the general visual response amplitude decrease during stimulation for each stimulated site. *Contacts of interest (i.e., pool of face-selective contacts outside of stimulation) are color-coded according to the baseline-corrected amplitude difference between the average of Pre1 and Pre2 and the stimulation periods (stimulation effect; PreGA-Stim). Contacts with a significant difference are circled in red (z-score>2.32, p < 0.01).*

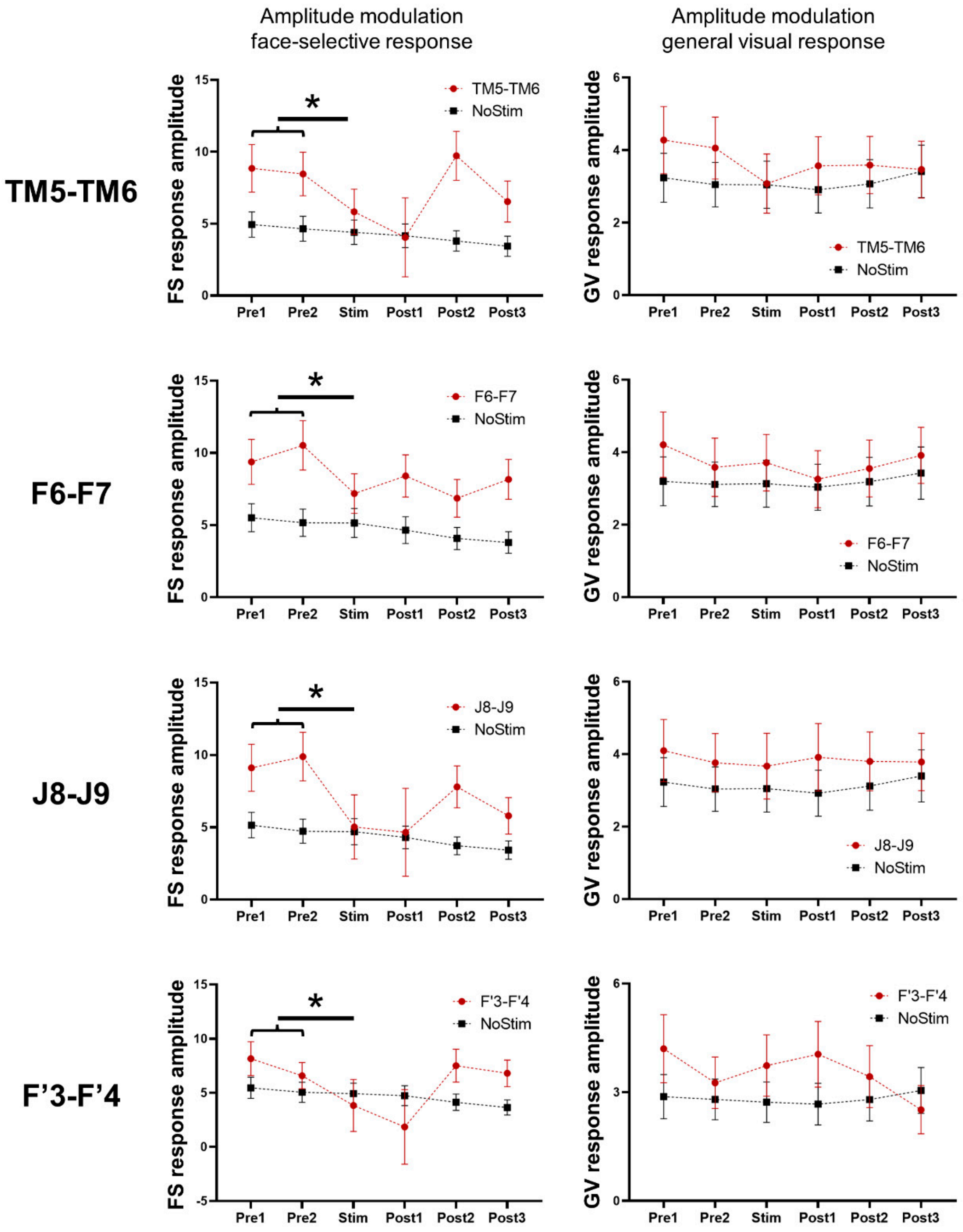

Amplitude modulation face-selective response
Amplitude modulation general visual response
TM5-TM6
F6-F7
J8-J9
F'3-F'4
FS response amplitude
GV response amplitude
NoStim
Pre1
Pre2
Stim
Post1
Post2
Post3
*

Figure S4. Global variation of the baseline-corrected amplitude of the face-selective (FS) and general visual (GV) response observed throughout the FPVS sequences. *Mean baseline-corrected amplitude of the face-selective and general visual response recorded throughout the FPVS sequence across the pool of contacts of interest (i.e., showing a significant face-selective response already outside stimulation, N=61 minus the 2 stimulated contacts) during the stimulated (Stim F6-F7, J8-J9, TM5-TM6, F'3-F'4) and non-stimulated (NoStim) sequences. (*) indicates a significant difference between the amplitude decrease (i.e., PreGA-Stim) observed in the stimulated sequences and the one observed in the non-stimulated sequences (p < .05; 2-tailed paired permutation test).*

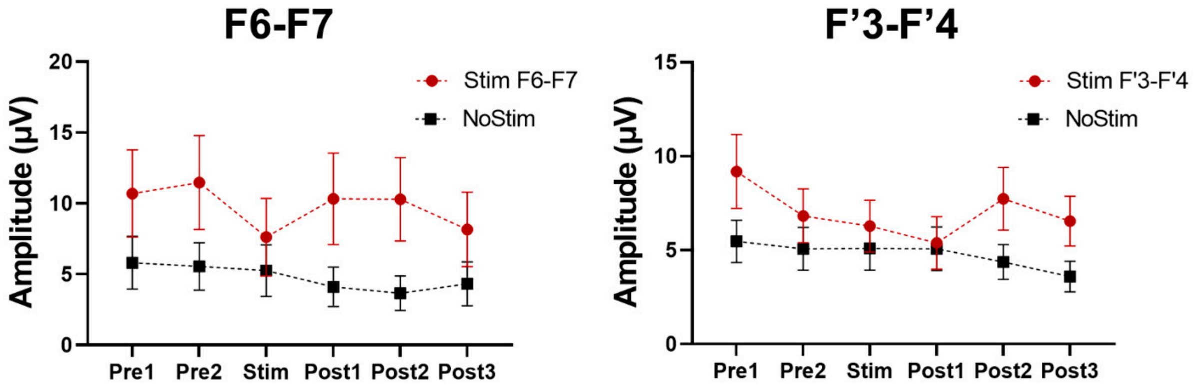

Figure S5. Comparison of the effect evoked by the stimulation of the right and left LatFG throughout the FPVS sequences. *Mean baseline-corrected amplitudes of the face-selective response throughout the FPVS sequence across the pool of contacts of interest (i.e., contacts that show a significant face-selective response outside stimulation and are located in the opposite hemisphere to the one stimulated; N=18 for stim F6-F7 and N=43 for stim F'3-F'4) during the stimulated (Stim F6-F7, F'3-F'4) and non-stimulated (NoStim) sequences.*

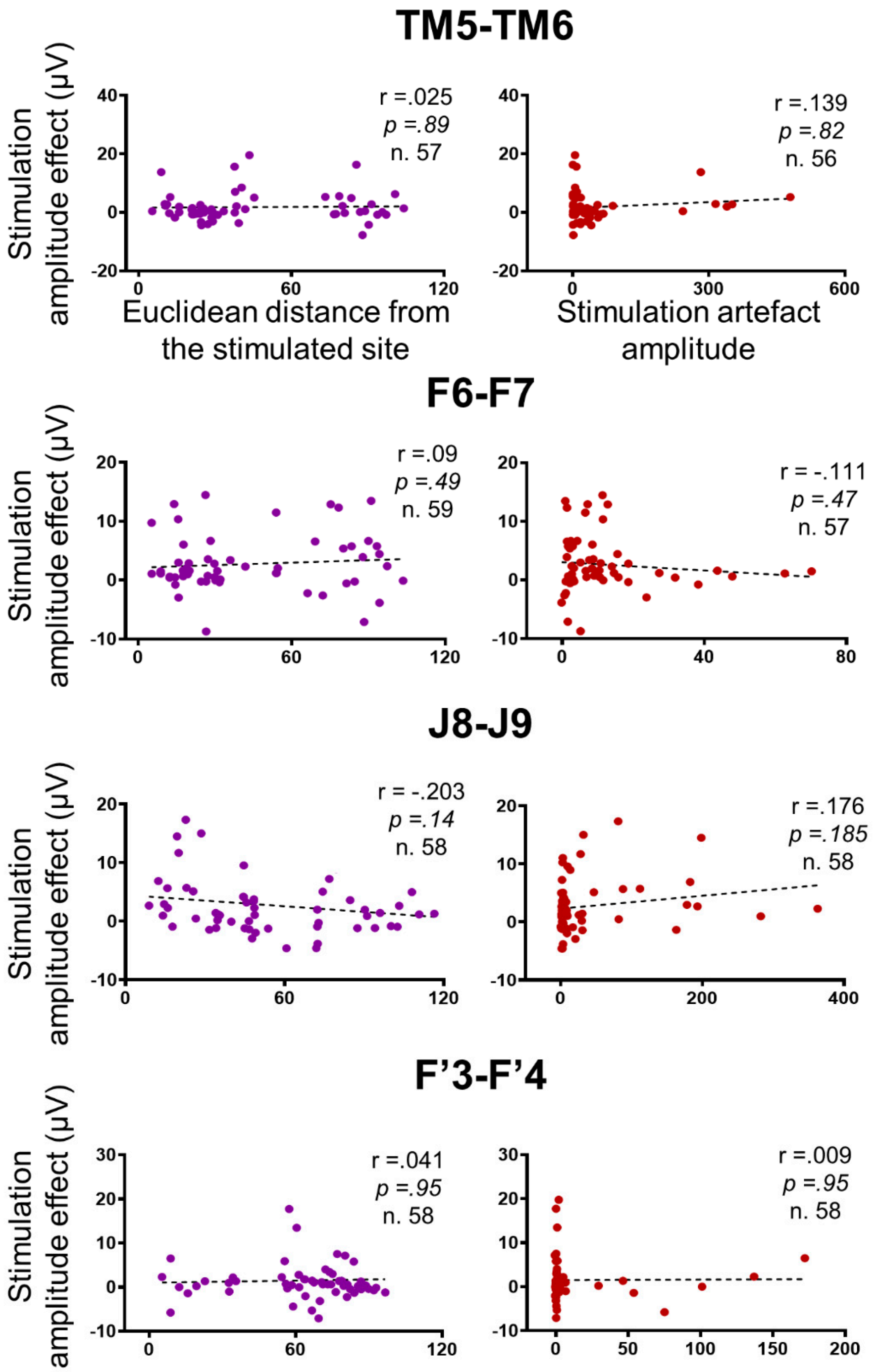

Figure S6. Correlation plots between the stimulation amplitude effect for the face-selective responses (baseline-corrected amplitude difference between the average of Pre1 and Pre2 and stimulation periods) and physical measurements (Euclidean distance from the stimulation site and the amplitude of the stimulation artefact) across the contacts of interest (N=59). *Outliers with values*

higher than z-score=3 were removed. The Pearson correlation coefficient, the p-values, and the number of contacts included in the analyses are indicated for each correlation. False Discovery Rate (FDR) corrections were applied to control for multiple comparisons [67]. Note that the amplitude of the stimulation artefact was quantified in the frequency domain by summing the first 17 harmonics of the 55Hz stimulation frequency (at standard and aliased frequencies).

Table S1*. *Behavioral performance of YR and 5 control subjects in face/object recognition tasks. *All tasks were administered through E-Prime 2.0 on a 60Hz screen positioned at about 60cm. We also tested five control participants (matched on gender, age, handedness, and educational level) with the same tests. To compare the YR's performance to the control participants, we used the modified t-test of Crawford and Howell for single-case studies [55] with a p-value < 0.05 (one-tailed) considered as statistically significant. Acc: Accuracy in percentages; RT = Response Times in ms; NA: Non-Applicable.*

**Cognitive tasks**			**Subject YR**	**Controls (n = 5)**	**t-test (Crawford-Howell)**
**Benton Facial Recognition Test Electronic version** [53]		**Acc (/54)**	34	45,6 ± 2,2	**t = -4.81, p = .004**
		**RT (ms)**	147	237,8 ± 57,4	t = -1.44, p = .11
**Face and car delayed matching** [54]	**CARS**	**Acc Upright (%)**	88,9	98,9 ± 1,4	**t = -6.52, p = .0014**
		**Acc Inverted (%)**	88,9	97,7 ± 1,1	**t = -7.3, p = .0009**
		**RT Upright (ms)**	1088	1165,8 ± 132,3	t = -0.54, p = .31
		**RT Inverted (ms)**	1216	1258,9 ± 113,4	t = -0.34, p = .37
		**Index**	5,5	4,5 ± 2,2	t = 0.41, p = .35
	**FACES**	**Acc Upright (%)**	77,8	93,3 ± 6	**t = -2.36, p = .04**
		**Acc Inverted (%)**	77,8	73,3 ± 6,7	t = 0.61, p = .29

		**RT Upright (ms)**	1487	1759 ± 106	t = -2.34, p = .04
		**RT Inverted (ms)**	1682	1972,5 ± 408,6	t = -0.65, p = .28
		**Index**	6,1	16,7 ± 10,2	t = -0.95, p = .2
**Famous and non-famous face simultaneous matching** [47]	**FAMOUS**	**Acc Upright (%)**	77,3	100 ± 0	NA
		**Acc Inverted (%)**	45,5	87,3 ± 5,3	**t = -7.2, p = .0009**
		**RT Upright (ms)**	2707	1735 ± 450,1	t = 1.97, p = .06
		**RT Inverted (ms)**	3468	3804,9 ± 1239,8	t = -0.25, p = .41
	**NON-FAMOUS**	**Acc Upright (%)**	81,8	97,3 ± 3,6	**t = -3.93, p = .009**
		**Acc Inverted (%)**	68,2	84,5 ± 7,9	t = -1.88, p = .07
		**RT Upright (ms)**	2946	2565,3 ± 617,6	t = 0.57, p = .3
		**RT Inverted (ms)**	3966	4342 ± 1218,5	t = -0.28, p = .39
**Face memory** [47]	**FAMOUS**	**Acc (%)**	81,8	98,2 ± 2,2	**t = -6.8, p = .001**
		**RT (ms)**	1840	1969,4 ± 770,1	t = -0.15, p = .44
	**NON-FAMOUS**	**Acc (%)**	54,5	92,7 ± 4,6	**t = -7.58, p = .0008**
		**RT (ms)**	1909	2175,1 ± 620,9	t = -0.39, p = .36
**Famous face pointing** [47]		**Acc (%)**	60,0	98,4 ± 2,3	**t = -15.2, p = .00005**
		**RT (ms)**	2385	1929 ± 503,6	t = 0.83, p = .23
**Famous name pointing** [47]		**Acc (%)**	98,0	100 ± 0	NA

	**RT (ms)**	1797	1563,6 ± 265,8	t = 0.8, p = .23

Table S2. Quantification of the stimulation effect for the face-selective (FS) and general visual (GV) responses (threshold z-score>2.32).

Stimulation site	N. of contacts with a significant amplitude decrease only for the FS response	N. of contacts with a significant amplitude decrease for both responses (FS and GV)	N. of contacts with a significant amplitude decrease only for the GV response
**TM5-TM6**<br>right AntFG	8	1	7
**F6-F7**<br>right LatFG	12	0	0
**J8-J9**<br>right IOG	10	2	1
**F'3-F'4**<br>left LatFG	4	0	5

Table S3. Quantification of the stimulation effect for the face-selective and general visual responses (threshold z-score>1.64).

Stimulation site	N. of contacts with a significant amplitude decrease only for the FS response	N. of contacts with a significant amplitude decrease for both responses (FS and GV)	N. of contacts with a significant amplitude decrease only for the GV response
**TM5-TM6**<br>right AntFG	13	1	9
**F6-F7**<br>right LatFG	18	4	2
**J8-J9**<br>right IOG	15	4	4
**F'3-F'4**<br>left LatFG	7	1	10
